# Leaf nitrogen and phosphorus resorption efficiencies are related to drought resistance across woody species in a Chinese savanna

**DOI:** 10.1093/treephys/tpad149

**Published:** 2023-12-15

**Authors:** Shu-Bin Zhang, Yu Song, Han-Dong Wen, Ya-Jun Chen

**Affiliations:** CAS Key Laboratory of Tropical Forest Ecology, Xishuangbanna Tropical Botanical Garden, Chinese Academy of Sciences, Mengla, Yunnan 666303, China; T-STAR Core Team, Xishuangbanna Tropical Botanical Garden, Chinese Academy of Sciences, Mengla, Yunnan 666303, China; Key Laboratory of Ecology of Rare and Endangered Species and Environmental Protection (Ministry of Education), Guangxi Normal University, Guilin, Guangxi 541004, China; CAS Key Laboratory of Tropical Forest Ecology, Xishuangbanna Tropical Botanical Garden, Chinese Academy of Sciences, Mengla, Yunnan 666303, China; T-STAR Core Team, Xishuangbanna Tropical Botanical Garden, Chinese Academy of Sciences, Mengla, Yunnan 666303, China; Yuanjiang Savanna Ecosystem Research Station, Xishuangbanna Tropical Botanical Garden, Chinese Academy of Sciences, Yuanjiang, Yunnan 653300, China; CAS Key Laboratory of Tropical Forest Ecology, Xishuangbanna Tropical Botanical Garden, Chinese Academy of Sciences, Mengla, Yunnan 666303, China; T-STAR Core Team, Xishuangbanna Tropical Botanical Garden, Chinese Academy of Sciences, Mengla, Yunnan 666303, China; Yuanjiang Savanna Ecosystem Research Station, Xishuangbanna Tropical Botanical Garden, Chinese Academy of Sciences, Yuanjiang, Yunnan 653300, China

**Keywords:** leaf construction cost, nutrient resorption efficiency, seasonal drought

## Abstract

Leaf nutrient resorption and drought resistance are crucial for the growth and survival of plants. However, our understanding of the relationships between leaf nutrient resorption and plant drought resistance is still limited. In this study, we investigated the nitrogen and phosphorus resorption efficiencies (NRE and PRE), leaf structural traits, leaf osmotic potential at full hydration (Ψ_osm_), xylem water potential at 50% loss of xylem-specific hydraulic conductivity (P_50_) and seasonal minimum water potential (Ψ_min_) for 18 shrub and tree species in a semiarid savanna ecosystem, in Southwest China. Our results showed that NRE and PRE exhibited trade-off against drought resistance traits (Ψ_osm_ and P_50_) across woody species. Moreover, this relationship was modulated by leaf structural investment. Species with low structural investment (e.g., leaf mass per area, leaf dry mass content and leaf construction cost [LCC]) tend to have high NRE and PRE, while those with high LCCs show high drought resistance, showing more negative Ψ_osm_ and P_50_.These results indicate that species with a lower leaf structural investment may have a greater need to recycle their nutrients, thus exhibiting higher nutrient resorption efficiencies, and vice versa. In conclusion, nutrient resorption efficiency may be a crucial adaptation strategy for coexisting plants in semiarid ecosystems, highlighting the importance of understanding the complex relationships between nutrient cycling and plant survival strategies.

## Introduction

Leaf nutrient resorption is a physiological process by which plants withdraw nutrients before leaf shedding and transfer them to other living tissues or storage organs (Vitousek 1982). Therefore, nutrient resorption is a crucial component of nutrient conservation strategies, and is pivotal for ecosystem nutrient cycling, plant growth, reproduction and other physiological processes (Killingbeck 1996, Yuan and Chen 2009, Freschet et al. 2010, Vitousek et al. 2010, Vergutz et al. 2012). Previous studies have shown that increased nutrient resorption contributes to plant growth (Zhang et al. 2015, Sartori et al. 2022). Characterizing nutrient resorption patterns and identifying their drivers help us to understand plant life-history strategies and fitness. Moreover, drought stress exerts significant selective pressure on the life-history strategies of tree species in tropical seasonal forests and savannas (Choat et al. 2012, 2018, Guillemot et al. 2022), and physiological drought resistance is crucial for plant survival during long periods of low soil water availability (Anderegg et al. 2019). Despite decades of research on nutrient resorption, the link between nutrient resorption and plant drought resistance remains elusive.

Nutrient resorption efficiency (NuRE) is characterized by the percentage reduction in nutrients between green leaves and senesced leaves (Killingbeck 1996). N (Nitrogen) and P (phosphorus) are two essential nutrients that limit plant growth and development (Aerts and Chapin 2000, He et al. 2020). On average, N resorption efficiency (NRE) and P resorption efficiency (PRE) across global woody plants are 62.1% and 64.9%, respectively (Vergutz et al. 2012); however, NRE and PRE vary widely among biomes and species (Yuan and Chen 2009, He et al. 2020). Precipitation, temperature and nutrient availability are the determining factors for NRE and PRE at the biome scale (Pugnaire and Chapin 1993, Vergutz et al. 2012, He et al. 2020, Wei et al. 2020, Urbina et al. 2021). In general, NRE increases while PRE decreases with decreasing mean annual temperature and precipitation (Yuan and Chen 2009). The concentrations of N (N_sen_) and P (P_sen_) in senesced leaves, which are termed as nutrient proficiency, can also be used to evaluate the level to which nutrients can be reduced in senesced leaves (Killingbeck 1996). Previous studies have showed that N_sen_ and P_sen_ were negatively associated with the leaf nutrient conservation traits (Killingbeck 1996, Zhang et al. 2015), namely, the low values of N_sen_ and P_sen_ indicated efficient nitrogen and phosphorus recycling and high NuRE (Aerts 1996). At the species scale, both nutrient resorption and proficiency are associated with leaf anatomical and resource economics traits (Freschet et al. 2010, Zhang et al. 2015, H. Chen et al. 2021). For instance, Zhang et al. (2015) found that NRE was positively correlated with leaf vein density and leaf thickness (LT) across dipterocarp tree species, which affected the relative growth rate of plants; in contrast, N_sen_ was negatively related to leaf vein density, leaf mass per area (LMA) and LT. Therefore, NuRE can be used to indicate plant ‘slow–fast’ performance and conservative–acquisitive strategies (Zhang et al. 2015, Sartori et al. 2022). Previous studies have suggested that different combinations of traits (leaf, stem and roots) can characterize the fast–slow plant economics spectrum, which shifts from fast to slow resource acquisition and conservation as the result of environmental selective force, and evolutionary and biophysical constraints (Wright et al. 2004, Reich 2014). It has been suggested that leaf traits are a good indicator of life-history strategies and plant performance (Poorter and Bongers 2006, Zhang et al. 2015). An increase in LMA and LT indicates high leaf structural investments and long leaf life span, which in turn enhances nutrient residence time in a leaf (Zhang et al. 2015). Therefore, in this study, we expected NuRE to be closely related to leaf structural investments.

Plants in tropical seasonal forests and savannas are exposed to periodic or extreme drought events. Common methods for estimating plant resistance to drought include measuring water potential at 50% loss of xylem-specific hydraulic conductivity (P_50_), leaf water potential at the turgor loss point (Ψ_tlp_) or leaf osmotic potential at full hydration (Ψ_osm_) (Choat et al. 2012, 2018, Bartlett et al. 2012*b*, 2014, Zhu et al. 2018, Yan et al. 2020). P_50_ is widely used as a proxy to assess (Choat et al. 2012), as it provides insights into the physiological thresholds to hydraulic impairment during drought (Anderegg et al. 2016, 2019). P_50_ is correlated with canopy dieback and drought-induced mortality during drought events (Y.J. Chen et al. 2021, McDowell et al. 2018, Anderegg et al. 2019). Previous studies have shown that Ψ_osm_ can be used as a rapid alternative to predict Ψ_tlp_ and is a powerful trait for predicting drought resistance and the plant distribution of species and biomes (Bartlett et al. 2012*a*, 2012*b*). Additionally, drought stress can be quantified as the minimum water potential during drought or dry periods (Ψ_min_) (Bhaskar and Ackerly 2006). A global synthesis has revealed a significant correlation between Ψ_min_ and P_50_ for both angiosperms and gymnosperms, indicating that drought resistance is associated with the level of drought stress experienced by the plants (Choat et al. 2012). Ψ_min_, as a hub trait, drives functional trade-offs and growth performance under drought stresses (Bartlett et al. 2016, Pratt et al. 2021, S.B. Zhang et al. 2022). Nutrient resorption and drought resistance are both associated with the overall plant physiological function and growth performance of plants (Bartlett et al. 2016, Sartori et al. 2022). However, no previous study has investigated the relationship between leaf nutrient resorption and drought resistance.

Savannas, which are characterized by high biodiversity and net primary productivity, are a significant component of terrestrial vegetation and are predominantly found in the tropical semiarid regions of Africa, Australia, India, South America and Southeast Asia (Grace et al. 2006). In Southwest China, the river valleys among the high mountains experience a hot-dry climate, and valley-type savannas are distributed in these valleys (Jin and Ou 2000, Zhu et al. 2020). In these savannas, both evergreen and deciduous species coexist, with evergreen species exhibiting drought resistance and deciduous species employing avoidance hydraulic strategies (Zhang et al. 2007, 2017, Y.J. Chen et al. 2021*b*). Seasonal drought exerts significant selective pressure, driving divergence in the hydraulic, photosynthetic and biomechanical traits of woody species with contrasting leaf phenologies in these Chinese savannas (S.B. Zhang et al. 2022). However, the classification of species into phenology groups, such as evergreen, deciduous and brevi-deciduous, may be somewhat arbitrary (Bucci et al. 2004, Goldstein et al. 2008). The continuous variations in eco-physiological traits provide a more comprehensive representation of the ecological complexity and adaptations of tropical savanna species to their seasonal environment (Goldstein et al. 2008). Additionally, drought stress may reduce NuRE. For instance, in a subtropical monsoon forest, NRE and PRE in *Lithocarpus glaber* experienced a substantial decrease of 56.5% and 53.8% in NRE and PRE, respectively, due to summer drought (Xu et al. 2020). Similarly, both chronic and intense drought significantly decreased species-level NuRE in a temperate steppe (Liang et al. 2022). Moreover, drought stress may limit nutrient resorption from senesced leaves, resulting in more nutrient loss compared with leaves that are normally defoliated leaves (Marchin et al. 2010). Nevertheless, it remains unclear whether seasonal drought also influences the continuous variation in nutrient resorption for these savanna species.

In the study, we investigated the N and P concentrations in green and senesced leaves, leaf morphological and structural traits, leaf construction cost (LCC), Ψ_osm_, P_50_ and Ψ_min_ of 18 tree and shrub species in a savanna ecosystem located in Southwest China. Our primary focus was to analyze the relationships between NuRE, drought resistance traits and leaf structural traits. Previous studies have demonstrated that species with a shared ancestry tend to have similar functional traits, suggesting that plant traits may be influenced by evolutionary history (Blomberg et al. 2003, Morand and Poulin 2003, Losos 2008). However, no previous study has directly tested whether nutrient resorption, hydraulic trait and leaf structural traits show significant phylogenetic signal for Chinese savanna species. To account for phylogenetic effects on traits, we also examined the phylogenetic signals and evolutionary correlations among functional traits. By doing so, we aimed to provide a better understanding of the complex relationships between nutrient resorption, drought resistance and leaf structural traits in Chinese savanna species. In this study, we aimed at addressing the following hypotheses.

(i) We hypothesize that NuRE will exhibit a negative relationship with leaf structural investments, such as LMA, leaf dry matter content (LDMC) and LCC; in contrast, drought resistance will be positively related to these structural investments. On the other hand, we expect drought resistance to be positively correlated with these structural investments. A lower investment in LMA indicates a higher return on a given investment, which may result in a greater need to recycle nutrients (Westoby 1998). Conversely, plant tissues with higher construction costs may possess thicker mesophyll cell walls and a greater proportion of cell wall mass per unit dry mass (Wright and Cannon 2001, Onoda et al. 2017), thus enhancing plant drought resistance (Bartlett et al. 2016, Zhu et al. 2018).

(ii) Species with low drought resistance will tend to have high NRE and PRE, enabling efficient nitrogen and phosphorus recycling. These species often adopt a drought-avoidant strategy characterized by the presence of short-lived and physiologically active leaves, promoting high short-term productivity and rapid growth (Wright and Westoby 2003). The drought-vulnerable species may have a high need to reabsorb from senescent leaves to reproductive and storage organs, and regrow a new canopy (Gonzalez-Rebeles et al. 2021).

## Materials and methods

### Study site

The experiment was conducted at the Yuanjiang Savanna Ecosystem Research Station (23° 27′ 56″ N, 102° 10′ 40″ E, elevation 481 m a.s.l.), Chinese Academy of Sciences, located in Yuanjiang County, Yunnan Province, Southwest China. This research station was situated within Yuanjiang National Reserve. The climate of this study site is characterized as hot and dry, with a rainy season from May to October, followed by a dry season from November to April. Based on meteorological data of this station covering the period of 2012–21, the mean annual temperature was 25.0 °C, and the mean annual precipitation was 681.4 mm. Approximately 80% of the precipitation falls during the rainy season. Supplementary data in Figure S1 available as Supplementary data at *Tree Physiology* online, provide more detailed information. The savanna ecosystem at the research site is predominantly composed of rock outcrops, covering around 60–70% of the area, with a shallow soil layer. The soils in this region are classified as ferralic cambisol according to the Food and Agriculture Organization of the United Nations (FAO). The pH values in the 0–20 cm soil layer range from 6.63 to 7.75. The total N, P and K concentrations in soils are 3.96, 1.30 and 12.72 mg g^−1^, respectively, and the available N, P and K concentrations are 206.9, 13.28 and 576.3 mg kg^−1^, respectively (Y.B. Zhang et al. 2022). The soil ammonia nitrogen and nitrate nitrogen were 12.9 ± 0.9 and 2.5 ± 1.1 mg kg^−1^ in the rainy season, respectively; the soil ammonia nitrogen and nitrate nitrogen were 7.0 ± 1.0 and 1.9 ± 0.7 mg kg^−1^ in the dry season, respectively (Li et al. 2018). This particular savanna vegetation is typical of the hot and dry valleys found in the high mountains of Southwest China. This savanna vegetation has been protected since 1980s, though occasional mild human disturbances, such as grazing and fire, have been reported (Zhang et al. 2012). The dominant tree species in this savanna ecosystem are *Lannea coromandelica*, *Polyalthia cerasoides* and *Haldina cordifolia*.

### Plant material and leaf sampling

For this study, we selected 10 tree species and 8 shrub species based on the species composition of a long-term monitoring plot (Table 1). Among our sampled species, 5 are evergreen, while the remaining 13 are deciduous species. Evergreen species maintain a full canopy throughout the year while deciduous species experience a leafless period for at least one or more months during the dry season. In August during the peak of the rainy season, we collected the current-season, fully expanded, light-exposed mature and healthy green leaves for the measurements of leaf structural traits, leaf-water relations, nutrient concentrations, and caloric values. Depending on the leaf or leaflet size, we collected at least five leaves or leaflets from three plants per species. During the dry season, we collected the senescent leaves from the same individuals that green leaves were collected during the rainy season. Thus, both green and senescent leaves were sampled from three individuals per species. It is important to note that the senesced leaves were collected directly from the plants rather than from the litter during the dry season. Following the methods described by Zhang et al. (2015), we were able to easily identify the senesced leaves, which became detached by gently flicking the branches due to the forming of an abscission layer at the base of the petiole. The collection of senescent leaves was conducted according to the specific leaf senescence period of each species, taking into account the differences in leaf senescence patterns among species.

**Table 1:** List of species, family, leaf habits, growth forms, height and diameter at breast height (DBH) in the present study.

Species	Code	Family	Phenology	Growth forms	Height (m)	DBH (cm)
*Haldina cordifolia*	Hc	Rubiaceae	Deciduous	Tree	6.2 ± 0.4	41.9 ± 3.0
*Cipadessa cinerascens*	Cc	Meliaceae	Deciduous	Shrub	3.2 ± 0.2	7.4 ± 0.4
*Polyalthia cerasoides*	Pc	Annonaceae	Deciduous	Tree	6.3 ± 1.0	18.1 ± 1.1
*Vitex negundo*	Vn	Verbenaceae	Deciduous	Shrub	2.3 ± 0.2	3.6 ± 0.1
*Trigonostemon tuberculatum*	Tt	Euphorbiaceae	Deciduous	Shrub	2.5 ± 0.1	5.1 ± 0.3
*Bauhinia brachycarpa*	Bb	Leguminosae	Deciduous	Shrub	2.8 ± 0.33	3.2 ± 0.1
*Lannea coromandelica*	Lc	Anacardiaceae	Deciduous	Tree	5.7 ± 0.4	27.6 ± 1.9
*Terminalia franchetii*	Tf	Combretaceae	Deciduous	Tree	6.0 ± 0.1	8.0 ± 0.5
*Trema angustifolia*	Ta	Ulmaceae	Deciduous	Tree	5.6 ± 0.1	11.7 ± 2.2
*Strophioblachia fimbricalyx*	Sf	Euphorbiaceae	Deciduous	Shrub	2.5 ± 0.1	2.6 ± 0.1
*Terminthia paniculata*	Tp	Anacardiaceae	Deciduous	Shrub	3.3 ± 0.6	4.9 ± 1.2
*Woodfordia fruticosa*	Wf	Lythraceae	Deciduous	Shrub	3.3 ± 0.6	4.9 ± 1.2
*Campylotropis delavayi*	Cd	Leguminosae	Deciduous	Tree	2.3 ± 0.2	5.8 ± 0.2
*Tarenna depauperata*	Td	Rubiaceae	Evergreen	Shrub	2.3 ± 0.2	5.4 ± 0.1
*Pistacia weinmanniifolia*	Pw	Anacardiaceae	Evergreen	Tree	4.8 ± 0.2	5.1 ± 0.5
*Diospyros yunnanensis*	Dy	Ebenaceae	Evergreen	Tree	4.2 ± 0.4	6.3 ± 0.4
*Carissa spinarum*	Cs	Apocynaceae	Evergreen	Tree	3.0 ± 0.1	6.8 ± 0.9
*Burretiodendron kydiifolium*	Bk	Malvaceae	Evergreen	Tree	3.3 ± 0.4	5.1 ± 0.8

### Leaf structural traits

In the field, we collected the current-season, fully expanded, light-exposed green leaves or leaflets and placed them in plastic bags within a sampling cooler. Subsequently, we transported the samples to the nearby laboratory. Upon arrival at the laboratory the leaves or leaflets were scanned using a 300-d.p.i. resolution scanner (National Institutes of Health, Bethesda, MD, USA). The digital photographs obtained from the scanner were then analyzed using ImageJ software to determine leaf size (LS, cm^2^). In addition to LS, we measured LT (mm) at the bottom, middle and top of each leaf or leaflet. The measurements of LT at different positions were averaged. To prepare the leaves for subsequent measurements, we immersed the fresh leaves or leaflets in distilled water until the water potential of all the rehydrated leaves were near zero (higher than −0.2 MPa), even for the leaves with latex. Afterwards, we carefully wiped the water on the leaf blade surface using a paper towel. Next, we determined the saturated mass (M_s_) of the leaves by weighing them on a balance (0.0001 g, Mettler-Toledo, AL204, Shanghai, China). To obtain the dry mass (M_d_), these fresh leaves or leaflets were oven-dried at 80 °C for 48 h, then weighted to obtain M_d_. Finally, LMA (g m^−2^) was calculated by dividing M_d_ by LS. LDMC (%) was calculated as the ratio of M_d_ to M_s_. Leaf density (LD, g cm^−3^) was calculated by dividing LMA by LT.

### Nutrient concentrations

The collected leaf samples were subjected to oven-drying at 80 °C for a minimum of 48 h. Subsequently, the dried leaves were finely ground using a crusher. The fine powder is used for further measurements of nitrogen and phosphorus concentrations. To determine the nitrogen concentrations in the green leaves (N_gr_, g kg^−1^) and senesced leaves (N_sen_, g kg^−1^), we employed a C–N elemental analyzer (Vario MAX CN, Elementar Analysensysteme GmbH, Hanau, Germany). For the analysis of phosphorus concentrations, other samples were digested with 8 mL HNO_3_ (65–68%) and 4 mL HClO_4_ (70–72%). Following digestion, the samples were dissolved in 4 mL HCl (3 M). The P concentrations in green leaves (P_gr_, g kg^−1^) and senesced leaves (P_sen_, g kg^−1^) were determined using an inductively coupled plasma atomic emission spectrometer (iCAP 7400, Thermo Fisher Scientific, Bremen, Germany). To calculate NuRE (NRE and PRE), the following formula was utilized (Killingbeck 1996, Vergutz et al. 2012):


$$ \mathrm{RE}=\left(1-\frac{{\mathrm{Nu}}_{\mathrm{sen}}}{{\mathrm{Nu}}_{\mathrm{gr}}}\mathrm{MCLF}\right)\ast 100\% $$

where Nu_sen_ and Nu_gr_ represent the nutrient concentrations on a mass basis in senesced and green leaves, respectively, and MCLF is the mass loss correction factor. The MCLF is calculated as the ratio of the average dry mass of senesced and green leaves (Vergutz et al. 2012).

### Leaf construction cost

To analyze the gross caloric value (GCV, kJ g^−1^) of the samples, we utilized an oxygen bomb calorimeter (C5000, IKA, Germany). The samples were combusted at a temperature of 550 °C for a duration of 4 h. Following combustion, the ash concentration (AS, g g^−1^) was determined based on the dry mass of each sample. The ash-free caloric value (AFCV, kJ g^−1^) was then calculated using the formula: GCV/(1 – AS/100). This calculation takes into account the AS to determine the caloric value of the sample without the contribution of the ash content. Furthermore, we calculated the LCC (g glu g^−1^) using the following formula (Williams et al. 1987):


$${} \mathrm{LCC}=\frac{\left(0.06968\times \mathrm{AFCV}-0.065\right)\times \left(1-\mathrm{AS}\right)+7.5\times \left(\mathrm{K}\times{\mathrm{N}}_{\mathrm{gr}}/14.0067\right)}{0.89} $$

where AFCV is the ash-free caloric value, AS is the ash concentration, N_gr_ is the N concentration in green leaves and K represents the oxidation state of the N source (+5 for nitrate or −3 for ammonium). In Chinese savannas, ammonium is the main source of soil N (Li et al. 2018), so for this study, we assumed *K* = −3 in this study.

### Leaf osmotic potential at full hydration

The branches with mature, healthy leaves were collected at predawn during the rainy season. To maintain leaf hydration, the samples were wrapped in black plastic and transported in a sampling box to the laboratory at the Yuanjiang Savanna Ecosystem Research Station. In the laboratory, the terminal branches were re-cut under distilled water and rehydrated. During rehydration, we closely monitored the rehydrated leaves or leaflets until the leaf water potential was near zero, even for the leaves with latex. Next, we sampled discs from leaves or leaflets collected on each branch using a 6-mm-diameter punch. These discs were wrapped in foil and immediately placed in liquid nitrogen for a minimum duration of 5 min to rapidly freeze the samples. We used sharp-tipped tweezers to puncture the frozen discs at least 20 times quickly. The leaf osmotic potential at leaf full turgor (Ψ_osm_) was determined using a vapor pressure osmometer chamber at 25 °C (VAPRO 5600, WESCOR Co., Logan, UT, USA).

### Xylem water potential at 50% loss of xylem-specific hydraulic conductivity

During the rainy season, the terminal branches with leaves were collected from selected individuals for each species at predawn. The following methods were employed to determine stem hydraulic conductivity and vulnerability to cavitation. Firstly, we determined the maximum vessel length (MVL) using the air injection method developed by Brodribb and Field (2000). Stem segments longer than the MVL were used to determine stem hydraulic conductivity. To measure stem vulnerability curves, we used the air-pressurization method as described by Cochard et al. (1992). The stem segments were flushed with KCl solution at a pressure of 100 kPa for a minimum of 30 min to remove air embolisms. Subsequently, we determined the maximum hydraulic conductivity (*K*_max_) for each stem segment. Next, the above segments were placed inside a pressure chamber with both ends protruding (PMS1505D-EXP, PMSS Instruments, Albany, OR, USA). The pressure inside the chamber was raised to 0.5 MPa and maintained for at least 10 min. It was then reduced to a basal level of 0.01 MPa to allow for equilibration. This process was repeated, incrementally raising the injection pressure by 0.5 or 1.0 MPa each time until the stems were fully cavitated. The residual pressure inside the chamber was maintained to ensure that no refilling could occur during measurements. At each pressurization step, the percentage loss of xylem-specific hydraulic conductivity (PLC, %) was calculated using the formula: PLC = 100% × (*K*_max_ – *K*_i_)/*K*_max_. *K*_i_ represents the hydraulic conductivity at a given pressurization step. Finally, we fitted a sigmoid function to fit the vulnerability curve using the equation developed by Pammenter and Vander Willigen (1998): 


$$ \mathrm{PLC}=100/(1+\exp(a\times(\Psi_{\mathrm{x}}-\Psi_{\text{0}}))) $$

where *a* is the maximum slope of the curve, and *Ψ*_0_ is the xylem water potential at 50% loss of hydraulic conductivity (P_50_).

### Minimum seasonal water potential

During the dry season from November 2014 to April 2015, we monitored the dynamics of midday water potential for the sampled species. The leaf-bearing branches of evergreen species and leafless terminal twigs of deciduous species were wrapped with black plastic bags and aluminum in the evening before the measurements. For the evergreen species, we used the leaves and for the deciduous species, we used the leafless terminal twigs to evaluate the seasonal minimum water potential (Ψ_min_) of the plant species between 12:00 and 14:00 h. We considered the most negative midday water potential during dry periods as Ψ_min_ (S.B. Zhang et al. 2022). This approach allowed us to investigate the water potential of the plants under drought conditions, which is crucial for understanding their ability to resist drought stress.

### Phylogenetic tree

Fresh leaf tissues from the 18 species included in this study were collected in September 2021. Total DNA was extracted from fresh leaf tissue using the modified hexadecyltrimethylammonium bromide procedure and treated with RNase A (Sigma). Primers designed for polymerase chain reaction (PCR) amplification were used to obtain the *rbcL* sequences from genomic DNA following the method described by Dong et al. (2014). The PCR amplification was performed at 94 °C for 30 s, 55 °C for 40 s and 72 °C for 1 min, with 36 cycles, followed by a final extension at 72 °C for 10 min. The obtained *rbc*L gene sequences were aligned using Clustal X (Aiyar 2000). The nucleic acid substitution model was calculated using Model Generator v0.84, and the optimal model of ‘GTR + F + R3’ was selected. To reconstruct the phylogenetic relationships among the 18 studied species, the maximum-likelihood method was employed using RAxML 7.2.6 (Stamatakis 2014). One thousand bootstrap replicates were performed to obtain node support and confidence in the phylogenetic tree. Finally, a phylogenetic tree of the 18 studied species was constructed (Figure S2 available as Supplementary data at *Tree Physiology* online).

### Statistical analyses

All statistical analyses were performed using R (version 4.0.2, R Core Team 2020). The Shapiro–Wilk test was used to evaluate the normality of all traits (Patrick 1982), and those that did not satisfy normality were log-transformed (Table S1 available as Supplementary data at *Tree Physiology* online). Higher absolute values represented higher leaf drought resistance (−Ψ_osm_) or xylem drought resistance (–P_50_), or stronger drought exposure (−Ψ_min_). To facilitate data analysis, the values of Ψ_min_, Ψ_osm_ and P_50_ were converted from negative to positive for further analysis. The units were represented as ‘–MPa’ (Table 2). Linear mixed models (LMMs) were employed to test the effects of phenology (evergreen and deciduous) and growth-form (tree and shrub) on the traits. In the models, phenology and growth-form were treated as fixed effects, while species are considered as random effect. To account the effects of phylogeny to trait associations, phylogenetic principal component analysis (PPCA) was used to evaluate the multivariate associations of trait contrasts using the ‘*FactoMinerR*’ package (Lê et al. 2008). Pearson correlation analysis was employed to test the bivariate relationships between pairs of traits.

**Table 2:** Functional traits, abbreviations, units and their mean values and ranges examined in this study.

Variables	Abbreviation	Unit	Min	Mean	Max
Leaf size	LS	cm^2^	3.2	59.7	268.8
Leaf thickness	LT	mm	0.16	0.23	0.31
Leaf mass per area	LMA	g m^−2^	25.6	74.7	149.6
Leaf density	LD	g cm^−3^	0.13	0.33	0.64
Leaf dry mass content	LDMC	%	20.3	32.3	44.3
Leaf construction cost	LCC	g glu g^−1^	1.23	1.42	1.58
Nitrogen concentration in green leaves	N_gr_	g kg^−1^	11.4	24.8	40.5
Phosphorus concentration in green leaves	P_gr_	g kg^−1^	0.67	1.37	2.68
Nitrogen concentration in senesced leaves	N_sen_	g kg^−1^	5.6	13.6	28.9
Phosphorus concentration in senesced leaves	P_sen_	g kg^−1^	0.32	0.74	2.10
Nitrogen resorption efficiency	NRE	%	19.6	51.2	82.3
Phosphorus resorption efficiency	PRE	%	22.2	52.9	79.9
Leaf water potential at turgor loss point	Ψ_osm_	−MPa	0.88	1.95	3.01
Minimum seasonal water potential	Ψ_min_	−MPa	1.38	3.15	4.58
Water potential at 50% loss of xylem-specific hydraulic conductivity	P_50_	−MPa	1.78	2.95	4.08
Maximum vessel length	MVL	cm	23.7	51.0	75.2

The phylogeny signal of all traits was assessed using Blomberg’s *K* statistic. A *K* value close to 1 indicates a significant phylogenetic effect, while a value close to 0 suggests no phylogenetic signal (Blomberg et al. 2003). Additionally, phylogenetically independent contrast (PICs) was also applied to evaluate the correlations between pairs of traits, taking into account the phylogenetic effect. This analysis was performed using the ‘*APE*’ and ‘*Picante*’ packages (Kembel et al. 2010, Paradis and Schliep 2019).

## Results

The 18 sampled woody species showed wide variations in leaf structural traits, construction cost, nitrogen and phosphorus concentrations in green and senesced leaves, and hydraulic traits varied significantly among the 18 sampled woody species (Table 2, Table S2 available as Supplementary data at *Tree Physiology* online). Furthermore, the results of the phylogenetic signal test revealed that only LCC and N_gr_ exhibited a significant phylogenetic signal (*P* < 0.05, Table S3 available as Supplementary data at *Tree Physiology* online). In contrast, the values of *K* were <1 and corresponding *P*-values were >0.05 for the other traits, suggesting a lack of phylogenetic conservatism.

The results of LMMs revealed that phenology had a significant effect on LMA, LD, LDMC, NRE, Ψ_osm_, Ψ_min_ and P_50_ (Table 3, *P* < 0.05); however, no significant differences between evergreen and deciduous groups were found for other traits (*P* > 0.05). Additionally, the effects of growth-forms (tree and shrub) were significant only for Ψ_min_, but not significant for other traits (Table 3).

**Table 3:** Effects of phenology and growth-form on the traits using LMM. Trait abbreviations are shown in Table 1. Phenology and growth-form were fixed effects, and species are random effects. Significance (*P* < 0.05) was shown in bold. Trait abbreviations are the same as in Table 2.

Traits	Phenology		Growth-form	
	*F*	*P*	*F*	*P*
LS	0.780	0.370	0.041	0.841
LT	0.868	0.351	0.027	0.871
LMA	11.045	**0.001**	0.799	0.376
LD	7.682	**0.006**	0.628	0.432
LDMC	5.017	**0.028**	0.155	0.695
LCC	3.268	0.050	1.026	0.316
N_gr_	4.276	0.087	0.843	0.363
P_gr_	5.209	0.054	0.712	0.403
N_sen_	0.043	0.797	2.918	0.094
P_sen_	0.760	0.684	2.579	0.115
NRE	5.407	**0.019**	0.427	0.516
PRE	2.038	0.099	1.303	0.259
Ψ_osm_	4.783	**0.014**	2.810	0.100
Ψ_min_	12.596	**<0.001**	5.463	**0.024**
P_50_	15.660	**<0.001**	1.332	0.254
MVL	0.015	0.761	0.477	0.493

**Figure 1:**
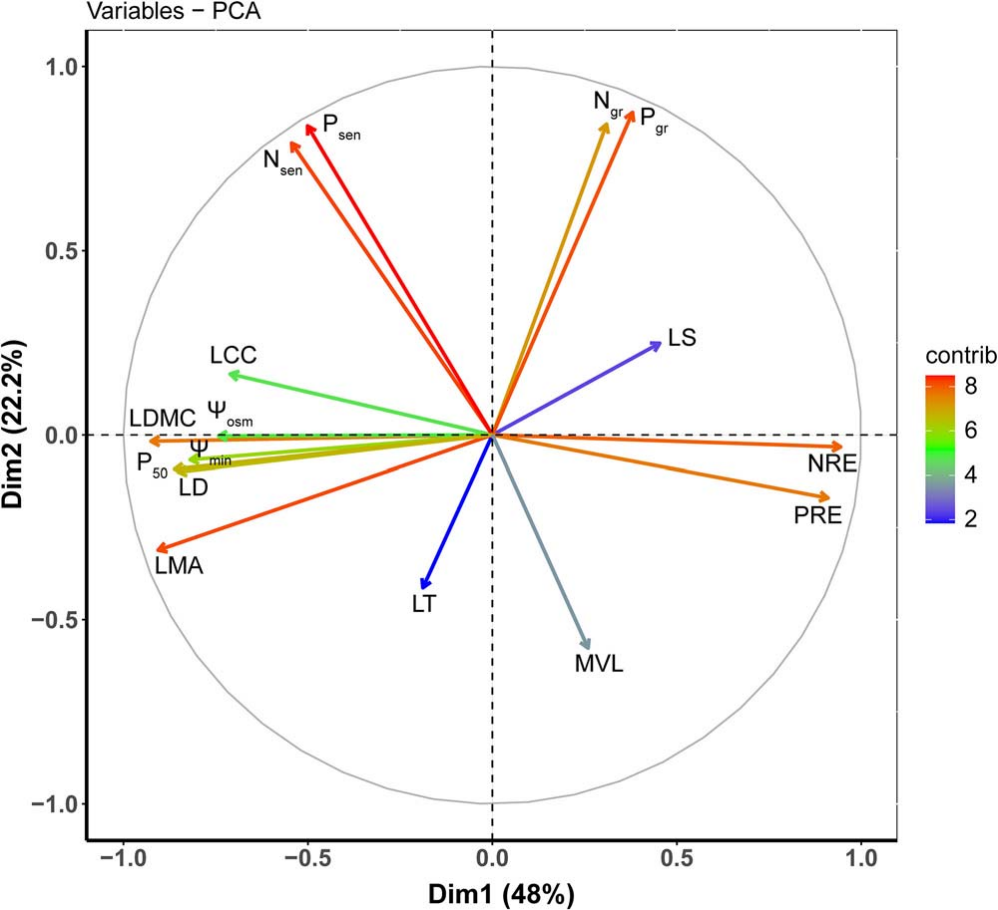
Phylogenetic principal component analysis conducted on 16 traits among 18 woody species in a savanna ecosystem, Southwest China. Trait abbreviations are the same as in Table 2.

The result of PPCA showed that the Axis 1 and Axis 2 explained 48.0% and 22.2% of the total variance, respectively (Figure 1). The negative side of Axis 1 was associated with leaf mass investment traits (e.g., LMA, LDMC, LD and LCC), leaf and stem drought resistance traits (e.g., P_50_ and Ψ_osm_), plant drought exposure (Ψ_min_). On the other hand, the positive side of Axis 1 was associated with nutrient resorption efficiencies (e.g., NRE and PRE). In addition, the positive side of Axis 2 was linked to nutrient concentrations of green and senescent leaves (N_gr_, P_gr_, P_sen_ and N_sen_), while the negative of Axis 2 was associated with MVL.

**Figure 2:**
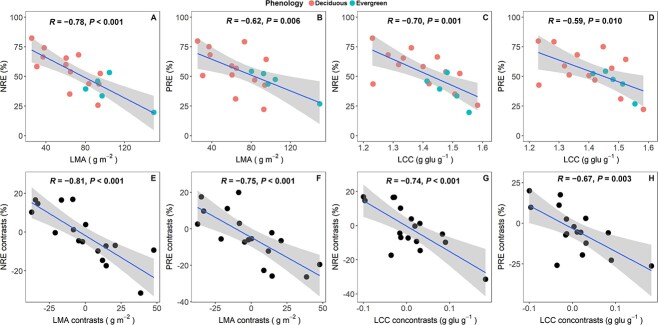
Pearson and PICs correlations between nutrient resorption efficiencies and leaf traits. The upper four panels (A–D) showed the Pearson correlation across 18 savanna woody species and the lower four panels (E–H) showed the PICs based on 17 contrasts. Trait abbreviations are the same as in Table 2.

The cross-species Pearson correlation analysis revealed that both NRE and PRE were negatively correlated with LMA and LCC (*P* < 0.05, Figure 2A–D) as well as with the other traits of leaf mass investments (LD and LDMC) (*P* < 0.05) (Table S4 available as Supplementary data at *Tree Physiology* online). Taking or without taking phylogeny into account, the correlations between NuRE and leaf structural traits were very similar (Figure 2, Table S4 available as Supplementary data at *Tree Physiology* online).

**Figure 3:**
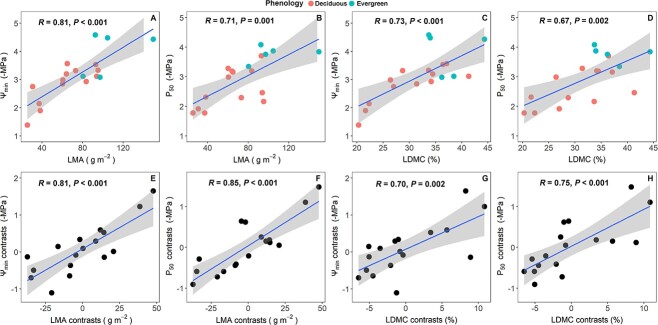
Pearson and PICs correlations between hydraulic traits and leaf traits the upper four panels (A–D) showed the Pearson correlation across 18 savanna woody species and the lower four panels (E–H) showed the PICs based on 17 contrasts. Trait abbreviations are the same as in Table 2.

**Figure 4:**
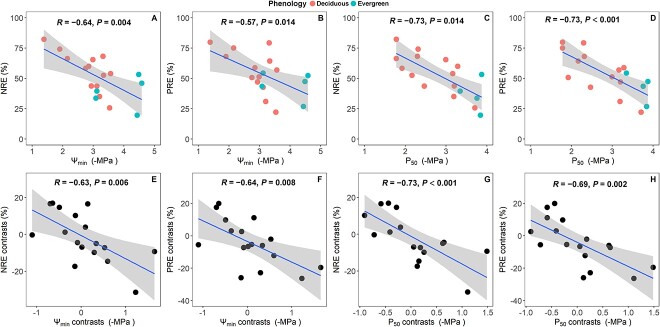
Pearson and PICs correlations between hydraulic traits and nutrient resorption efficiencies. The upper four panels (A–D) showed the Pearson correlation across 18 savanna woody species and the lower four panels (E–H) showed the PICs based on 17 contrasts. Trait abbreviations are the same as in Table 2.

Both Ψ_min_ and P_50_ were positively correlated with LMA and LDMC by taking or without taking phylogeny into account (Figure 3). Ψ_osm_ showed no correlation with LD and LDMC using Pearson correlation, but a significant relationship emerged using PICs (Table S4 available as Supplementary data at *Tree Physiology* online). Regarding the associations of NRE and PRE with drought-resistance traits, Ψ_min_ and P_50_ demonstrated significant correlations with NRE and PRE by taking or without taking phylogeny into account (Figure 4, *P* < 0.05). Furthermore, Ψ_osm_ was significantly related to PRE by taking or without taking phylogeny into account. The correlation between Ψ_osm_ and NRE was not significant using Pearson correlation; however, it reached significant when PICs was employed (Table S4 available as Supplementary data at *Tree Physiology* online).

## Discussion

Our findings demonstrate a negative relationship between both NRE and PRE and leaf structural investments (i.e., LMA, LDMC and LCC). This supports our initial hypothesis. Moreover, our results indicated that species with high drought resistance (as indicated by more negative values of Ψ_osm_ and P_50_) tend to exhibit low nutrient resorption efficiencies; in contrast, species with low drought resistance tend to have a greater need to recycle their nutrients, leading to higher nutrient resorption efficiencies. This finding supported our second hypothesis. Overall, our study reveals a negative association between leaf nitrogen and phosphorus resorption efficiencies and plant drought resistance among woody species in the Chinese savanna. Furthermore, this trade-off is modulated by leaf structural traits.

### Nutrient resorption and drought resistance traits are related to leaf structural traits

Our study revealed that species with low structural investment (e.g., LMA, LD, LDMC and LCC) tended to have high NRE and PRE. NuRE plays a crucial role in nutrient-use strategies, affecting plant growth performance and fitness, particularly in arid and nutrient-poor ecosystems (May and Killingbeck 1992, Urbina et al. 2021). In dry savanna ecosystems, tree species adopt rapid development strategies (Williams et al. 1997). Many savanna tree species, particularly the deciduous ones, are capable of quickly developing a full canopy of leaves either before or during the initial rainfall events by utilizing nutrient reserves stored in their storage organs (February and Higgins 2016). Consequently, increasing NRE and PRE could contribute to the efficient nutrient conservation and the rapid replenishment of nitrogen and phosphorus resources.

In contrast, our study revealed that drought-resistance traits (such as Ψ_osm_ and P_50_), plant drought exposure (Ψ_min_) and leaf structural traits (e.g., LMA, LD, LDMC and LCC) clustered together on the negative side of Axis 1 in the PPCA (Figure 1). This indicates the presence of covariant relationships between drought resistance traits and leaf structural investments. This study site is characterized by a dominant hot-dry climate with distinct rainy season (May–October) and dry season (November–April) (Figure S1 available as Supplementary data at *Tree Physiology* online). Additionally, this savanna is characterized by rock outcrops and shade soils, resulting in a lack of groundwater reserves (Y.B. Zhang et al. 2022). Drought stress serves as the primary selective pressure and is the most significant limiting factor that induced the mortality and dieback of woody species in this savanna (Y.J. Chen et al. 2021, Zhang et al. 2017). More negative values of P_50_ and Ψ_osm_ indicate greater stem and leaf drought resistance. However, this adaptation comes at a cost, namely, a higher fractions of cell wall mass per unit of dry mass (Ding et al. 2014, Onoda et al. 2017). In our study, the drought-resistant species, particularly those evergreen ones, exhibited enhanced leaf structural investments and maintained more negative leaf water potentials. This is especially crucial for sustaining hydraulic conductance, stomatal conductance and photosynthetic carbon gain during the dry season (Blackman et al. 2010, Bartlett et al. 2012*b*, 2016).

### The NRE and PRE are negatively related to plant drought resistance

Our previous studies have indicated that the selection force exerted by seasonal drought for half of the year drives the continuous variations and trade-offs of eco-physiological traits among the Chinese woody species (Zhang et al. 2007, 2017, S.B. Zhang et al. 2022). In this study, we observed a continuum of leaf nutrient resorption (NRE and PRE), plant drought resistance and drought exposure (Ψ_min_) among the species examined. To the best of our knowledge, this is the first study to investigate the associations between nutrient resorption and plant drought-resistance traits.

Our findings indicate that species with less negative Ψ_min_ tend to have higher NRE and PRE, resulting in faster nutrient return in the Chinese savanna ecosystem. On the other hand, species with high drought exposure show low nutrient resorption. This continuum observed in nitrogen and phosphorus resorption is closely associated with plant drought exposure and strategies of nutrient utilization. There are several possible reasons for the involvement of drought resistance in nutrient resorption. Species with dehydration avoidance strategies, such as deciduous species with less negative Ψ_osm_ and P_50_, often exhibit low structural investment. This ‘fast-return’ strategy relies on maximizing nutrient recovery from senescent leaves (Kobe et al. 2005), thus explaining their efficient nutrient resorption. In contrast, woody species with drought resistance strategies commonly exhibit higher leaf construction and maintenance costs, as well as long-lived leaves (Williams et al. 1989, Liu et al. 2022). These structural traits enable woody species to maintain greater drought resistance (more negative Ψ_osm_ and P_50_) throughout extended periods of seasonal drought, thereby experiencing more negative Ψ_min_ (Bartlett et al. 2016, Zhu et al. 2018). This conservative strategy reflects efficient nutrient conservation and aligns with a ‘slow’ leaf economic spectrum (Wright et al. 2004). Therefore, our findings demonstrate that in the hot and dry Chinese savanna habit, woody species exhibit a ‘fast–slow’ continuum, ranging from rapid nutrient turnover coupled with low drought resistance to a conservative strategy prioritizing high drought resistance.

## Conclusions

In summary, we examined the associations between NuRE, drought resistance traits and leaf structural traits in woody species within a Chinese savanna characterized by pronounced seasonal drought. Our findings revealed that species with lower drought resistance (indicated by negative values of Ψ_osm_ and P_50_) tended to exhibit higher NRE and PRE across woody species studied. Moreover, leaf structural investment played a role in modulating the trade-offs between plant drought resistance and nutrient resorption efficiencies. These results suggest that species with a lower leaf structural investment have a greater need to recycle their nutrients and demonstrate more efficient nutrient resorption. Both NuRE and drought resistance traits are crucial for plant growth and fitness. Therefore, considering the combined use of nutrient resorption and drought resistance may provide valuable insights into how plants adapt to their habitats. It is important to note that further studies are needed to investigate similar trait associations across a broader range of plant groups and habitats. Additionally, exploring how these relationships impact life-history strategies and plant fitness would be an interesting avenue for future research.

## Supplementary Material

Supplementary_data_tpad149

## Data Availability

The data supporting this study are provided as supplementary data. Additionally, interested individuals may request the R code for data analysis from the corresponding author.
