# Supplementary material for: Leaf nitrogen and phosphorus resorption efficiencies are related to drought resistance across woody species in a Chinese savanna
Source: Tree Physiol. 2023 Dec 15;44(1):tpad149. doi: 10.1093/treephys/tpad149 (PMC10849754; doi:10.1093/treephys/tpad149)
Supplement: Supplementary_data_tpad149 [file supplementary_data_tpad149.docx]

Supplementary data

**
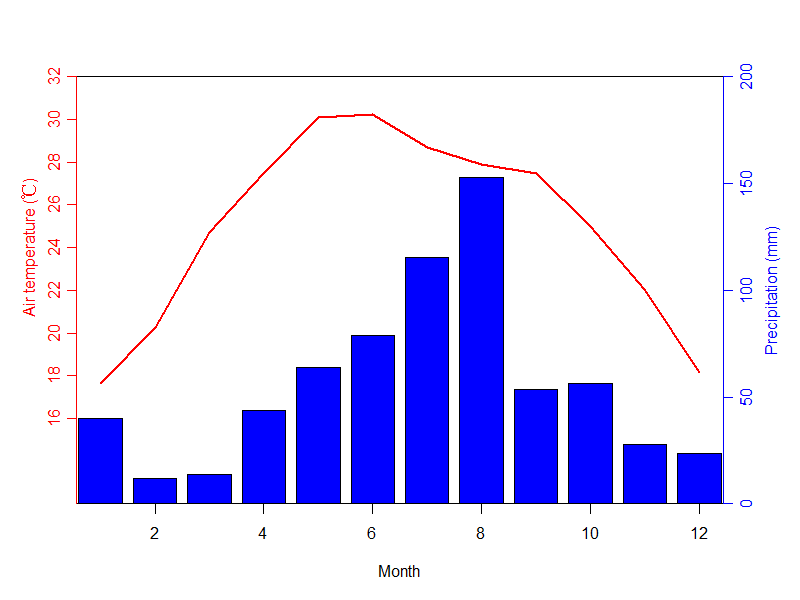
**

**Figure. S1.** Climate diagram for the period 2012-2021 in Yuanjiang savanna ecosystem research station. Red lines and blue bars represent monthly air temperature and precipitation, respectively.

**
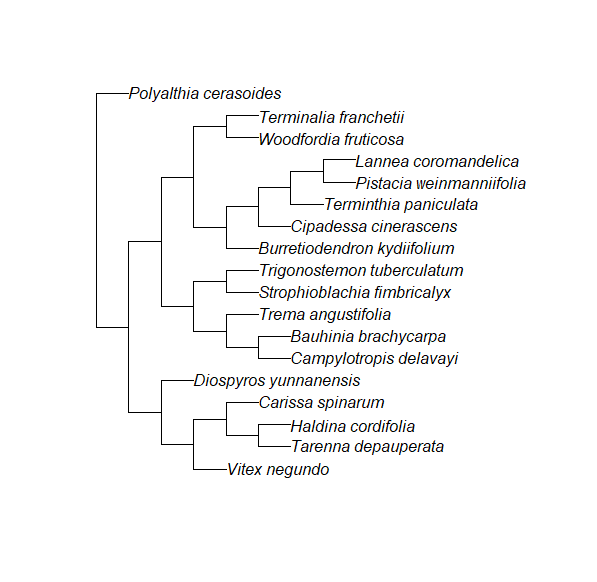
**

**Figure. S2.** Phylogenetic tree of the 18 savanna species studied in this study.**Table S1.** Results of Shapiro-Wilk normality test for all traits in this study. The statistic (*W*) and corresponding significance level in Shapiro-Wilk normality test were given. Significance (*P* < 0.05) was underlined and shown in bold. Trait abbreviations are the same as in Table 2.

| **Abbreviation** | **Unit** | ***W*** | ***P*-value** |
| --- | --- | --- | --- |
| LS | cm^2^ | 0.753 | **< 0.001** |
| LT | mm | 0.935 | 0.24 |
| LMA | cm^2^ g^-1^ | 0.947 | 0.38 |
| LD | g cm^-3^ | 0.949 | 0.40 |
| LDMC | % | 0.957 | 0.54 |
| LCC | g glu g^−1^ | 0.956 | 0.53 |
| N_gr_ | g kg^−1^ | 0.955 | 0.51 |
| P_gr_ | g kg^−1^ | 0.889 | **0.04** |
| N_sen_ | g kg^−1^ | 0.909 | 0.08 |
| P_sen_ | g kg^−1^ | 0.726 | **< 0.001** |
| NRE | % | 0.988 | 0.99 |
| PRE | % | 0.966 | 0.72 |
| Ψ_osm_ | -MPa | 0.938 | 0.27 |
| Ψ_min_ | -MPa | 0.940 | 0.29 |
| P_50_ | -MPa | 0.921 | 0.14 |
| MVL | cm | 0.969 | 0.78 |

**Table S2.** Individual values of measured traits in this study. Trait abbreviations and their units are the same as in Table 2.

| Species | individual | LS | LT | LMA | LD | LDMC | LCC | N**_gr_** | P**_gr_** | N**_sen_** | P**_sen_** | NRE | PRE | Ψ_osm_ | Ψ**_min_** | P_50_ | MVL | MCLR |
| --- | --- | --- | --- | --- | --- | --- | --- | --- | --- | --- | --- | --- | --- | --- | --- | --- | --- | --- |
| *Haldina_cordifolia* | 1 | 260.1 | 0.36 | 40.1 | 0.12 | 21.2 | 1.446 | 25.17 | 1.53 | 10.22 | 0.47 | 67.4 | 75.5 | 1.89 | 2.10 | 1.37 | 43.4 | 80.4 |
| *Haldina_cordifolia* | 2 | 257.8 | 0.27 | 33.2 | 0.12 | 21.1 | 1.448 | 24.28 | 1.47 | 10.64 | 0.47 | 64.8 | 74.1 | 1.71 | 2.35 | 1.85 | 50.3 | 80.2 |
| *Haldina_cordifolia* | 3 | 288.4 | 0.30 | 38.0 | 0.14 | 24.7 | 1.450 | 23.39 | 1.41 | 9.57 | 0.43 | 67.1 | 75.5 | 1.82 | 2.00 | 2.11 | 49.3 | 80.3 |
| *Cipadessa_cinerascens* | 1 | 22.0 | 0.18 | 68.6 | 0.39 | 34.0 | 1.508 | 36.55 | 2.42 | 27.71 | 2.05 | 37.7 | 30.4 | 1.61 | 3.35 | 3.19 | 20.8 | 82.1 |
| *Cipadessa_cinerascens* | 2 | 15.5 | 0.16 | 65.0 | 0.42 | 35.3 | 1.507 | 37.33 | 2.55 | 28.04 | 2.07 | 35.6 | 30.4 | 1.58 | 2.80 | 3.43 | 26.9 | 85.8 |
| *Cipadessa_cinerascens* | 3 | 13.5 | 0.16 | 58.8 | 0.38 | 34.1 | 1.506 | 38.11 | 2.68 | 31.04 | 2.18 | 32.2 | 32.2 | 1.60 | 3.45 | 2.95 | 23.4 | 83.3 |
| *Polyalthia_cerasoides* | 1 | 40.9 | 0.21 | 58.8 | 0.28 | 35.3 | 1.479 | 28.66 | 1.76 | 16.08 | 0.96 | 51.9 | 53.3 | 1.82 | 3.50 | 3.01 | 39.3 | 85.7 |
| *Polyalthia_cerasoides* | 2 | 35.3 | 0.22 | 67.1 | 0.31 | 37.9 | 1.477 | 29.53 | 1.81 | 16.10 | 0.96 | 52.9 | 54.2 | 1.66 | 3.50 | 3.35 | 39.1 | 86.3 |
| *Polyalthia_cerasoides* | 3 | 43.6 | 0.23 | 68.6 | 0.31 | 38.1 | 1.475 | 30.4 | 1.86 | 16.21 | 0.85 | 56.9 | 63.1 | 1.99 | 3.70 | 3.12 | 42.4 | 80.7 |
| *Vitex_negundo* | 1 | 92.1 | 0.24 | 92.8 | 0.39 | 33.2 | 1.524 | 23.99 | 1.35 | 13.44 | 0.57 | 55.7 | 66.8 | 1.64 | 3.45 | 2.40 | 85.9 | 79.0 |
| *Vitex_negundo* | 2 | 98.9 | 0.23 | 97.8 | 0.42 | 34.2 | 1.524 | 24.13 | 1.45 | 12.99 | 0.56 | 54.8 | 67.8 | 1.61 | 3.35 | 2.05 | 76.0 | 83.9 |
| *Vitex_negundo* | 3 | 94.7 | 0.23 | 95.2 | 0.42 | 33.6 | 1.523 | 24.27 | 1.55 | 14.17 | 0.71 | 47.1 | 58.5 | 1.61 | 3.20 | 2.04 | 63.6 | 90.6 |
| *Trigonostemon_tuberculatum* | 1 | 88.9 | 0.31 | 65.8 | 0.21 | 27.6 | 1.338 | 30.55 | 1.52 | 13.02 | 0.84 | 63.4 | 52.7 | 2.61 | 2.70 | 2.08 | 33.1 | 86.0 |
| *Trigonostemon_tuberculatum* | 2 | 93.6 | 0.33 | 66.6 | 0.20 | 27.6 | 1.334 | 33.15 | 1.59 | 12.63 | 0.81 | 68.3 | 57.4 | 3.02 | 3.05 | 3.20 | 33.5 | 83.2 |
| *Trigonostemon_tuberculatum* | 3 | 70.4 | 0.28 | 49.2 | 0.17 | 24.0 | 1.329 | 35.75 | 1.66 | 13.93 | 1.03 | 64.8 | 44.0 | 3.04 | 3.20 | 3.69 | 28.9 | 90.3 |
| *Bauhinia_brachycarpa* | 1 | 34.3 | 0.17 | 25.4 | 0.15 | 25.0 | 1.399 | 32.78 | 1.36 | 16.46 | 0.78 | 57.9 | 52.2 | 2.06 | 3.20 | 2.18 | 62.2 | 83.8 |
| *Bauhinia_brachycarpa* | 2 | 41.9 | 0.20 | 37.7 | 0.25 | 31.2 | 1.400 | 31.92 | 1.27 | 15.97 | 0.78 | 58.4 | 48.8 | 2.15 | 2.80 | 1.71 | 60.7 | 83.1 |
| *Bauhinia_brachycarpa* | 3 | 39.1 | 0.17 | 29.2 | 0.17 | 24.9 | 1.402 | 31.06 | 1.18 | 15.61 | 0.70 | 58.8 | 51.4 | 2.20 | 2.25 | 1.86 | 61.5 | 82.0 |
| *Lannea_coromandelica* | 1 | 90.5 | 0.18 | 26.9 | 0.15 | 20.6 | 1.231 | 25.69 | 1.7 | 5.76 | 0.37 | 84.0 | 84.5 | 0.79 | 1.35 | 1.76 | 39.7 | 71.3 |
| *Lannea_coromandelica* | 2 | 107.7 | 0.18 | 26.4 | 0.15 | 20.3 | 1.230 | 26.42 | 1.65 | 5.85 | 0.37 | 80.9 | 80.7 | 0.97 | 1.35 | 1.71 | 48.0 | 86.0 |
| *Lannea_coromandelica* | 3 | 71.7 | 0.15 | 23.4 | 0.15 | 20.0 | 1.229 | 27.15 | 1.6 | 5.32 | 0.44 | 81.9 | 74.5 | 0.87 | 1.45 | 1.86 | 54.3 | 92.6 |
| *Terminalia_franchetii* | 1 | 59.3 | 0.26 | 104.7 | 0.41 | 43.4 | 1.232 | 15.66 | 0.78 | 10.06 | 0.53 | 42.2 | 39.1 | 2.22 | 3.15 | 2.21 | 61.7 | 90.0 |
| *Terminalia_franchetii* | 2 | 66.6 | 0.21 | 77.5 | 0.37 | 37.5 | 1.232 | 15.89 | 0.82 | 10.53 | 0.53 | 43.9 | 45.7 | 2.47 | 3.15 | 2.62 | 58.0 | 84.7 |
| *Terminalia_franchetii* | 3 | 68.2 | 0.23 | 99.3 | 0.43 | 43.0 | 1.231 | 16.12 | 0.86 | 10.56 | 0.58 | 45.0 | 43.4 | 2.41 | 3.05 | 2.56 | 45.4 | 83.9 |
| *Trema_angustifolia* | 1 | 13.0 | 0.19 | 38.1 | 0.21 | 25.8 | 1.363 | 31.7 | 1.55 | 10.03 | 0.52 | 73.7 | 72.3 | 1.03 | 1.86 | 2.20 | 66.8 | 83.0 |
| *Trema angustifolia* | 2 | 12.0 | 0.19 | 40.9 | 0.22 | 20.4 | 1.360 | 32.9 | 1.48 | 9.42 | 0.53 | 75.1 | 69.0 | 0.92 | 1.88 | 2.34 | 76.1 | 86.9 |
| *Trema angustifolia* | 3 | 12.2 | 0.18 | 35.6 | 0.20 | 18.6 | 1.358 | 34.1 | 1.41 | 10.24 | 0.59 | 73.6 | 63.2 | 1.07 | 1.96 | 2.40 | 69.7 | 87.8 |
| *Strophioblachia_fimbricalyx* | 1 | 130.9 | 0.22 | 61.4 | 0.28 | 31.1 | 1.320 | 39.68 | 2.76 | 19.32 | 1.33 | 58.9 | 59.4 | 2.61 | 2.80 | 3.09 | 33.5 | 84.3 |
| *Strophioblachia_fimbricalyx* | 2 | 91.8 | 0.22 | 66.4 | 0.31 | 32.0 | 1.318 | 40.53 | 2.68 | 19.70 | 1.31 | 58.7 | 58.3 | 2.26 | 2.75 | 3.6 | 31.4 | 85.0 |
| *Strophioblachia_fimbricalyx* | 3 | 91.3 | 0.20 | 52.9 | 0.27 | 31.1 | 1.316 | 41.38 | 2.6 | 18.64 | 1.27 | 62.3 | 59.1 | 2.32 | 3.00 | 3.16 | 24.7 | 83.7 |
| *Terminthia_paniculata* | 1 | 33.0 | 0.23 | 104.8 | 0.46 | 39.2 | 1.582 | 16.8 | 0.75 | 13.94 | 0.73 | 26.9 | 14.4 | 1.79 | 3.55 | 4.39 | 40.0 | 88.1 |
| *Terminthia_paniculata* | 2 | 45.0 | 0.24 | 82.8 | 0.34 | 33.9 | 1.583 | 16.1 | 0.84 | 13.54 | 0.74 | 24.0 | 20.6 | 1.60 | 3.50 | 3.59 | 47.9 | 90.4 |
| *Terminthia_paniculata* | 3 | 40.9 | 0.23 | 91.1 | 0.40 | 36.1 | 1.585 | 15.4 | 0.93 | 14.07 | 0.79 | 26.3 | 31.5 | 1.50 | 3.55 | 3.14 | 54.2 | 80.7 |
| *Woodfordia_fruticosa* | 1 | 15.1 | 0.26 | 76.8 | 0.30 | 30.5 | 1.284 | 13.98 | 1.34 | 5.92 | 0.35 | 65.9 | 79.1 | 1.25 | 3.45 | 2.11 | 62.8 | 80.5 |
| *Woodfordia_fruticosa* | 2 | 22.3 | 0.24 | 80.0 | 0.34 | 28.8 | 1.282 | 14.68 | 1.27 | 6.48 | 0.36 | 64.0 | 77.2 | 1.28 | 3.25 | 2.31 | 59.0 | 81.5 |
| *Woodfordia_fruticosa* | 3 | 19.3 | 0.21 | 62.6 | 0.30 | 26.9 | 1.281 | 15.38 | 1.2 | 4.56 | 0.26 | 74.8 | 81.6 | 1.27 | 3.25 | 2.46 | 67.3 | 85.0 |
| *Campylotropis_delavayi* | 1 | 23.7 | 0.28 | 79.2 | 0.29 | 33.8 | 1.421 | 24.46 | 1.12 | 17.29 | 0.70 | 43.0 | 49.6 | 1.69 | 2.85 | 3.26 | 66.6 | 80.6 |
| *Campylotropis_delavayi* | 2 | 18.2 | 0.26 | 82.8 | 0.32 | 33.9 | 1.420 | 24.97 | 1.07 | 16.78 | 0.69 | 44.6 | 46.6 | 1.64 | 2.80 | 2.72 | 73.1 | 82.4 |
| *Campylotropis_delavayi* | 3 | 26.2 | 0.28 | 88.7 | 0.32 | 34.8 | 1.419 | 25.48 | 1.02 | 16.57 | 0.65 | 43.8 | 45.0 | 1.86 | 3.15 | 3.62 | 66.7 | 86.4 |
| *Tarenna_depauperata* | 1 | 63.3 | 0.29 | 109.4 | 0.38 | 34.0 | 1.481 | 28.09 | 0.83 | 15.77 | 0.50 | 54.1 | 50.4 | 2.90 | 4.70 | 4.21 | 59.1 | 81.8 |
| *Tarenna_depauperata* | 2 | 73.4 | 0.26 | 100.4 | 0.39 | 32.5 | 1.481 | 28.18 | 0.89 | 15.69 | 0.49 | 53.8 | 54.1 | 3.00 | 4.25 | 3.69 | 65.9 | 83.0 |
| *Tarenna_depauperata* | 3 | 61.2 | 0.28 | 103.9 | 0.37 | 35.3 | 1.481 | 28.27 | 0.95 | 14.94 | 0.65 | 52.1 | 38.0 | 3.12 | 4.50 | 3.72 | 58.8 | 90.6 |
| *Pistacia_weinmanniifolia* | 1 | 3.2 | 0.13 | 64.4 | 0.50 | 34.1 | 1.457 | 16.12 | 1.18 | 11.85 | 0.58 | 38.9 | 59.3 | 1.53 | 3.45 | 3.30 | 55.4 | 83.1 |
| *Pistacia_weinmanniifolia* | 2 | 3.6 | 0.18 | 91.8 | 0.58 | 41.5 | 1.456 | 16.96 | 1.11 | 11.36 | 0.56 | 42.6 | 56.4 | 1.41 | 3.10 | 3.66 | 57.5 | 85.7 |
| *Pistacia_weinmanniifolia* | 3 | 2.9 | 0.17 | 84.9 | 0.53 | 39.8 | 1.454 | 17.8 | 1.04 | 13.32 | 0.65 | 37.1 | 47.4 | 1.45 | 2.80 | 3.08 | 72.3 | 84.1 |
| *Diospyros_yunnanensis* | 1 | 7.2 | 0.24 | 145.5 | 0.60 | 44.3 | 1.556 | 12.39 | 0.63 | 11.99 | 0.57 | 11.7 | 17.5 | 2.89 | 4.25 | 3.86 | 39.1 | 91.2 |
| *Diospyros_yunnanensis* | 2 | 7.8 | 0.26 | 171.6 | 0.66 | 43.6 | 1.555 | 12.74 | 0.67 | 11.91 | 0.57 | 28.7 | 35.4 | 3.43 | 4.60 | 3.78 | 45.7 | 76.3 |
| *Diospyros_yunnanensis* | 3 | 6.5 | 0.20 | 131.7 | 0.65 | 45.1 | 1.554 | 13.09 | 0.71 | 13.10 | 0.63 | 18.6 | 27.8 | 2.66 | 4.45 | 3.89 | 33.1 | 81.4 |
| *Carissa_spinarum* | 1 | 10.8 | 0.30 | 99.6 | 0.27 | 35.3 | 1.515 | 10.69 | 0.78 | 8.77 | 0.54 | 24.7 | 36.9 | 2.81 | 3.10 | 3.98 | 46.9 | 91.8 |
| *Carissa_spinarum* | 2 | 9.9 | 0.29 | 87.3 | 0.31 | 36.9 | 1.514 | 11.42 | 0.83 | 9.11 | 0.53 | 34.8 | 48.2 | 2.54 | 3.10 | 3.34 | 43.4 | 81.7 |
| *Carissa_spinarum* | 3 | 10.7 | 0.34 | 104.5 | 0.31 | 36.4 | 1.512 | 12.15 | 0.88 | 9.37 | 0.63 | 41.6 | 45.7 | 2.96 | 3.05 | 3.95 | 56.7 | 75.8 |
| *Burretiodendron_kydiifolium* | 1 | 103.5 | 0.27 | 95.0 | 0.36 | 35.4 | 1.414 | 24.86 | 1.31 | 16.15 | 0.64 | 45.4 | 58.9 | 1.85 | 5.00 | 3.68 | 45.8 | 84.1 |
| *Burretiodendron_kydiifolium* | 2 | 85.7 | 0.26 | 103.5 | 0.40 | 34.1 | 1.414 | 25.19 | 1.25 | 15.76 | 0.65 | 45.4 | 54.3 | 1.50 | 4.40 | 4.14 | 45.5 | 87.2 |
| *Burretiodendron_kydiifolium* | 3 | 85.5 | 0.28 | 79.2 | 0.28 | 31.6 | 1.410 | 25.52 | 1.19 | 15.64 | 0.78 | 47.5 | 43.9 | 1.94 | 4.35 | 4.43 | 41.6 | 85.6 |

**Table S3.** Results of phylogenetic signal test of all the traits in this study. *K*-value is phylogenetic signal; *P* is the significance level. Significant phylogenetic signal was underlined and shown in bold (*P* < 0.05). Trait abbreviations are the same as in Table 2.

| **Abbreviation** | **Unit** | ***K-value*** | ***P*** |
| --- | --- | --- | --- |
| LS | cm^2^ | 0.308 | 0.667 |
| LT | mm | 0.415 | 0.178 |
| LMA | cm^2^ g^-1^ | 0.338 | 0.456 |
| LD | g cm^-3^ | 0.348 | 0.435 |
| LDMC | % | 0.455 | 0.597 |
| LCC | g glu g^−1^ | 0.626 | **0.041** |
| N_gr_ | g kg^−1^ | 0.542 | **0.030** |
| P_gr_ | g kg^−1^ | 0.436 | 0.263 |
| N_sen_ | g kg^−1^ | 0.349 | 0.605 |
| P_sen_ | g kg^−1^ | 0.454 | 0.292 |
| NRE | % | 0.317 | 0.605 |
| PRE | % | 0.258 | 0.946 |
| Ψ_osm_ | -MPa | 0.402 | 0.176 |
| Ψ_min_ | -MPa | 0.386 | 0.695 |
| P_50_ | -MPa | 0.315 | 0.814 |
| MVL | cm | 0.629 | 0.081 |

**Table S4.** Pearson’s correlations (lower left diagonal) and phylogenetically independent contrasts (PICs) (17 contrasts; upper right diagonal) among 16 functional traits. Trait abbreviations are the same as in Table 2. Coefficient values for significant correlations are underlined and shown in bold. Significant level: ^*^ 0.01< *P* < 0.05; ^**^0.001< *P* < 0.01; ^***^*P* < 0.001.

|  | **LS** | **LT** | **LMA** | **LD** | **LDMC** | **LCC** | **N_gr_** | **P_gr_** | **N_sen_** | **P_sen_** | **NRE** | **PRE** | **Ψ_osm_** | **Ψ_min_** | **P_50_** | **MVL** |
| --- | --- | --- | --- | --- | --- | --- | --- | --- | --- | --- | --- | --- | --- | --- | --- | --- |
| **LS** | — | 0.27 | -0.40 | -0.59^*^ | -0.41 | -0.33 | 0.37 | 0.21 | -0.04 | -0.03 | 0.39 | 0.21 | -0.15 | -0.23 | -0.40 | -0.16 |
| **LT** | 0.38 | — | 0.28 | -0.16 | 0.05 | 0.15 | -0.46 | -0.48^*^ | -0.20 | -0.19 | -0.20 | -0.20 | 0.24 | 0.21 | 0.31 | -0.12 |
| **LMA** | -0.34 | 0.31 | — | 0.86^***^ | 0.85^***^ | 0.53^*^ | -0.48^*^ | -0.55^*^ | 0.27 | 0.16 | -0.8^***^ | -0.71^**^ | 0.63^**^ | 0.81^***^ | 0.84^***^ | 0.01 |
| **LD** | -0.57^*^ | -0.18 | 0.85^***^ | — | 0.87^***^ | 0.51^*^ | -0.28 | -0.3 | 0.41 | 0.30 | -0.75^***^ | -0.61^**^ | 0.51^*^ | 0.71^**^ | 0.68^**^ | 0.05 |
| **LDMC** | -0.43 | 0.03 | 0.85^***^ | 0.87^***^ | — | 0.52^*^ | -0.32 | -0.33 | 0.50^*^ | 0.46 | -0.9^***^ | -0.78^***^ | 0.67^**^ | 0.70^**^ | 0.75^***^ | -0.14 |
| **LCC** | -0.29 | 0.15 | 0.51^*^ | 0.49^*^ | 0.47 | — | -0.16 | -0.14 | 0.55^*^ | 0.51^*^ | -0.74^***^ | -0.68^**^ | 0.28 | 0.59^*^ | 0.55^*^ | -0.18 |
| **N_gr_** | 0.39 | -0.26 | -0.58^*^ | -0.48^*^ | -0.48^*^ | -0.21 | — | 0.78^***^ | 0.53^*^ | 0.52^*^ | 0.39 | 0.19 | -0.19 | -0.16 | -0.25 | -0.23 |
| **P_gr_** | 0.28 | -0.36 | -0.55^*^ | -0.35 | -0.36 | -0.22 | 0.81^***^ | — | 0.44 | 0.49^*^ | 0.34 | 0.35 | -0.42 | -0.38 | -0.35 | -0.37 |
| **N_sen_** | 0.00 | -0.21 | 0.06 | 0.21 | 0.28 | 0.38 | 0.59^**^ | 0.54^*^ | — | 0.94^***^ | -0.54^*^ | -0.58^*^ | 0.32 | 0.44 | 0.40 | -0.44 |
| **P_sen_** | 0.00 | -0.23 | -0.04 | 0.12 | 0.23 | 0.29 | 0.63^**^ | 0.65^**^ | 0.92^***^ | — | -0.53^*^ | -0.60^**^ | 0.36 | 0.29 | 0.33 | -0.60^**^ |
| **NRE** | 0.47^*^ | -0.08 | -0.78^***^ | -0.79^***^ | -0.88^***^ | -0.70^**^ | 0.47^*^ | 0.38 | -0.38 | -0.33 | — | 0.90^***^ | -0.64^**^ | -0.64^**^ | -0.73^**^ | 0.30 |
| **PRE** | 0.34 | -0.05 | -0.62^**^ | -0.61^**^ | -0.73^**^ | -0.59^*^ | 0.19 | 0.27 | -0.52^*^ | -0.52^*^ | 0.89^***^ | — | -0.76^***^ | -0.62^**^ | -0.67^**^ | 0.40 |
| **Ψ_osm_** | 0.05 | 0.55^*^ | 0.54^*^ | 0.27 | 0.46 | 0.24 | -0.08 | -0.26 | 0.17 | 0.20 | -0.41 | -0.51^*^ | — | 0.62^**^ | 0.60^**^ | -0.40 |
| **Ψ_min_** | -0.15 | 0.28 | 0.81^***^ | 0.69^**^ | 0.73^**^ | 0.51^*^ | -0.27 | -0.34 | 0.32 | 0.17 | -0.64^**^ | -0.57^*^ | 0.53^*^ | — | 0.83^***^ | -0.05 |
| **P_50_** | -0.34 | 0.25 | 0.71^**^ | 0.59^*^ | 0.67^**^ | 0.52^*^ | -0.18 | -0.21 | 0.37 | 0.35 | -0.71^**^ | -0.69^**^ | 0.50^*^ | 0.76^***^ | — | -0.15 |
| **MVL** | -0.16 | -0.04 | 0.01 | 0.00 | -0.12 | -0.04 | -0.31 | -0.51^*^ | -0.45 | -0.63^**^ | 0.19 | 0.34 | -0.32 | -0.12 | -0.34 | — |
